# Enhancing organoid culture: harnessing the potential of decellularized extracellular matrix hydrogels for mimicking microenvironments

**DOI:** 10.1186/s12929-024-01086-7

**Published:** 2024-09-27

**Authors:** Chen Li, Ni An, Qingru Song, Yuelei Hu, Wenzhen Yin, Qi Wang, Yinpeng Le, Wenting Pan, Xinlong Yan, Yunfang Wang, Juan Liu

**Affiliations:** 1 Beijing International Science and Technology Cooperation Base for Antiviral Drugs, Beijing Key Laboratory of Environmental and Viral Oncology, College of Chemistry and Life Science, Beijing University of Technology, Beijing, 100124 China; 2 School of Clinical Medicine, Beijing Tsinghua Changgung Hospital, Hepato-Pancreato-Biliary Center, Tsinghua University, Beijing, 102218 China; 3 School of Clinical Medicine, Beijing Tsinghua Changgung Hospital, Clinical Translational Science Center, Tsinghua University, Beijing, 102218 China; 4 Key Laboratory of Digital Intelligence Hepatology (Ministry of Education/Beijing), School of Clinical Medicine, Tsinghua University, Beijing, 100084 China; 5 School of Materials Science and Engineering, Institute of Smart Biomedical Materials, Zhejiang Sci-Tech University, Hangzhou, 310018 China

**Keywords:** Decellularized Extracellular Matrix, Organoid culture, Hydrogels, Microenvironment, Natural-based biomaterials

## Abstract

Over the past decade, organoids have emerged as a prevalent and promising research tool, mirroring the physiological architecture of the human body. However, as the field advances, the traditional use of animal or tumor-derived extracellular matrix (ECM) as scaffolds has become increasingly inadequate. This shift has led to a focus on developing synthetic scaffolds, particularly hydrogels, that more accurately mimic three-dimensional (3D) tissue structures and dynamics in vitro. The ECM–cell interaction is crucial for organoid growth, necessitating hydrogels that meet organoid-specific requirements through modifiable physical and compositional properties. Advanced composite hydrogels have been engineered to more effectively replicate in vivo conditions, offering a more accurate representation of human organs compared to traditional matrices. This review explores the evolution and current uses of decellularized ECM scaffolds, emphasizing the application of decellularized ECM hydrogels in organoid culture. It also explores the fabrication of composite hydrogels and the prospects for their future use in organoid systems.

## Introduction

The extracellular matrix (ECM) is a sophisticated and intricate three-dimensional (3D) lattice synthesized by tissue-specific cells, providing both structural and biochemical scaffolding while orchestrating cellular dynamics within distinct microenvironments [1–3]. These specialized niches promote cellular proliferation and enhance intercellular communication [4]. Organoids, derived from 3D stem cell cultures, serve as precise tissue surrogates, mirroring the complex architecture and physiological functions of native tissues, and exhibit remarkable fidelity during serial passaging [5–7]. A pivotal breakthrough in organoid science occurred in 2009 with the pioneering establishment of intestinal organoid culture systems by Hans Clevers et al. [7], setting a new benchmark for subsequent organoid research endeavors.

Organoids, functioning as 3D models, surpass traditional 2D cultures in effectively mimicking the intricate histological, metabolic, and functional attributes of target organs [8–10]. The foundation of these models is based on the use of matrix scaffolds, with animal-derived hydrogels such as Matrigel and basement membrane extract (BME) playing an integral role [11]. Nevertheless, the clinical applicability of these scaffolds is hampered by their tumor-derived origins and batch-to-batch variability. While alternative scaffolds incorporating either natural or synthetic components, such as collagen and polyethylene glycol, have been explored, they have met with limited success [12, 13]. Recent advancements in decellularization techniques now enable the direct use of native ECM in organoid cultures, representing a significant step forward in the development of more physiologically relevant models [14, 15]. The development of dECM hydrogels has revolutionized organoid culture by providing a more biomimetic microenvironment that closely resembles the in vivo conditions. These dECM hydrogels, derived from native tissues, offer unparalleled advantages over traditional scaffolds, including enhanced biocompatibility, reduced immunogenicity, and the preservation of endogenous tissue-specific cues that are essential for guiding organoid development and function.

The native ECM comprises a complex assembly of structural proteins, growth factors, and proteoglycans, serving as a vital repository of bioactive cues that modulate cellular processes such as adhesion, morphogenesis, and signaling [2, 16, 17]. The transformation of decellularized ECM scaffolds (dECM) into hydrogels requires meticulous digestion and processing. This review examines recent breakthroughs in decellularization across diverse organs and provides an overview of the techniques used in the preparation of hydrogels. Furthermore, dECM hydrogels can be tailored to recapitulate the mechanical and biochemical properties of various tissues, allowing for the precise control over organoid microenvironments. This customization is vital for advancing the field of regenerative medicine and for creating more physiologically relevant models for disease research and drug discovery. By modifying component ratios or incorporating synthetic materials such as collagen or poly *N*-isopropylacrylamide, one can manipulate the mechanical properties of the hydrogel [18, 19]. Additionally, integrating cytokines and proteins can direct cellular growth and differentiation. These adaptations facilitate the simulation of disease states and the integration of composite hydrogels with advanced technologies such as 3D printing and microfluidics [3, 20–22]. Despite the acknowledged potential of natural extracellular matrices, the absence of standardized protocols for hydrogel evaluation presents a significant challenge. This paper examines methodologies to replicate organoid environments and enhance the application of dECM in organoid culture. It comprehensively reviews the intricacies of organoid culture requirements and the most recent advancements in the preparation and modification of acellular matrix hydrogels.

## Natural-based biomaterials: decellularized extracellular matrix

Simulating in vivo microenvironments constitutes a pivotal goal within the realm of organoid culture, aiming to accurately replicate the physiological functions of cells. dECM consists of biomaterials derived from human or animal organs or tissues, which are obtained through the application of decellularization techniques that eliminate immunogenic cellular components. Advances in decellularization have preserved the physicochemical and biological properties of the ECM, thus closely mimicking its native state. In three-dimensional cultures, dECM provides structural support as well as delivers essential biological signals.

### The history of dECM scaffolds

**Fig. 1:**
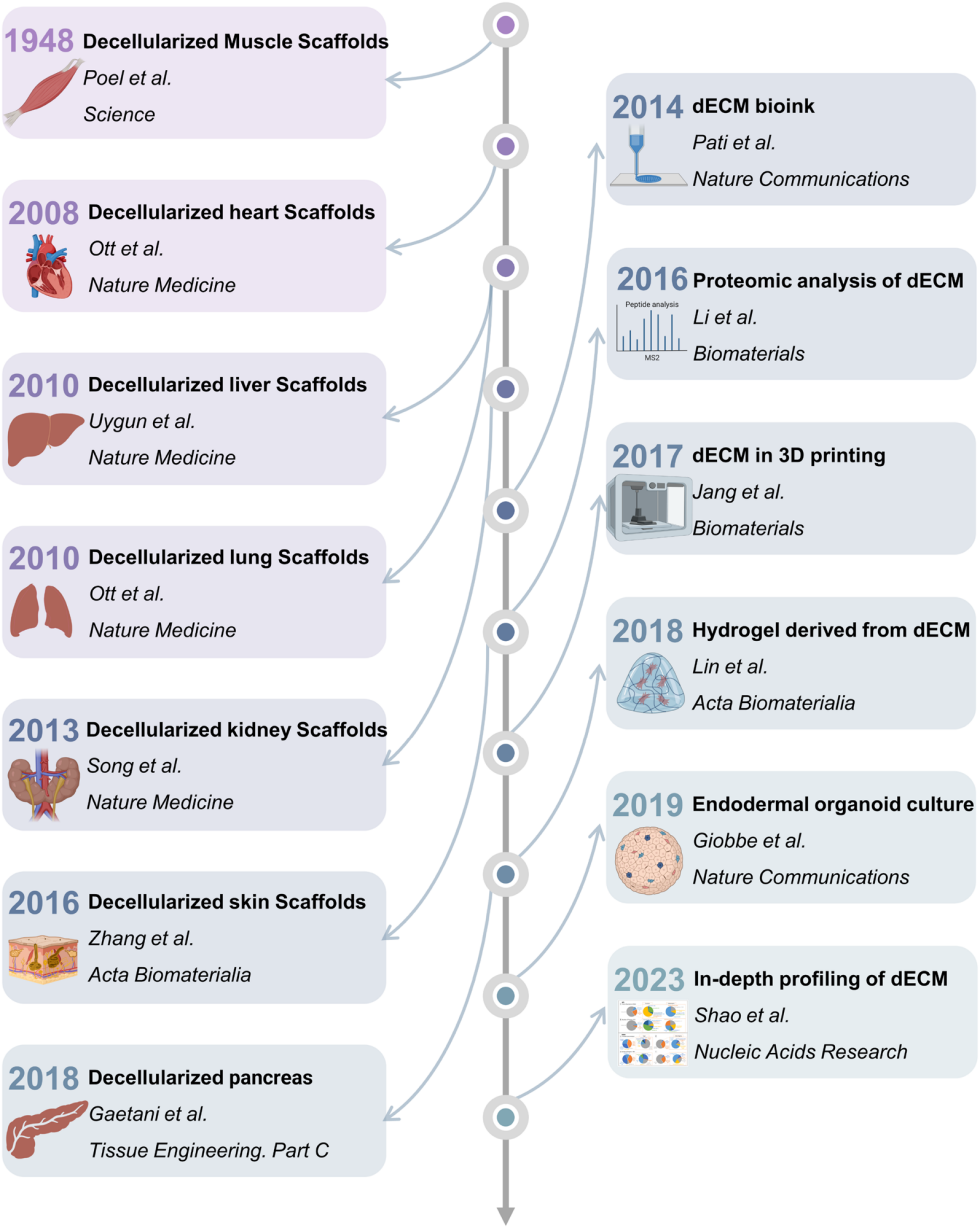
The history of dECM scaffolds. The emergence of decellularization technology dates back to 1948 [23], primarily applied to the basic decellularization of skeletal muscle. By 2008, this technique had been applied to the study of cardiac decellularization in murine models [26]. Subsequently, the creation of decellularized liver and lung scaffolds was accomplished in 2010 [28], followed by the development of decellularized kidney scaffolds in 2013, with orthotopic transplantation subsequently performed [29]. Studies into the use of dECM for skin and pancreatic tissues were initiated in 2016 [31] and 2018 [34], respectively. Since 2014, the use of dECM has become increasingly diverse. It was first transformed into bio-ink in 2014 [37], proteomic analysis of dECM was initiated in 2016 [39], 3D constructs were created using stem-cell-supported dECM bio-ink in 2017 [33], and dECM was converted into a hydrogel for further applications in 2018 [34]. In 2019, it was verified that dECM could also be used for organoid culture [36], and in 2023, by improving the sample preparation, mass spectrometry and bioinformatics methods, more comprehensive ECM proteins group information makes in-depth analysis on composition of ECM is possible [41]

Figure 1 provides an overview of the evolution of dECM scaffolds in the field of regenerative medicine. Since Poel’s foundational work on decellularization in 1948, researchers have thoroughly explored the use of dECM scaffolds across various human and animal organs [23]. In 1979, Hjelle et al. utilized sodium deoxycholate to isolate glomerular basement membranes, confirming their structural integrity through low-power transmission electron microscopy [24]. In 1995, Badylak et al. reported the successful repair of Achilles tendons in a canine model using xenogeneic small intestinal submucosa [25]. Subsequently, in 1998, Bader et al. developed a detergent-based method to decellularize porcine aortic valves, including RNase and DNase to remove potential cellular remnants [26].

The following decades heralded the expansion of research into whole-organ dECM. In 2008, Ott and colleagues initiated the decellularization of hearts via coronary perfusion using detergents, producing decellularized structures with perfusable vasculature and preserved chamber geometries [27]. By 2010, Uygun and associates had decellularized hepatic matrices, creating transplantable scaffolds and marking a significant advancement in the fabrication of recellularized liver scaffolds for transplantation support. These recellularized grafts retained liver-specific functions, such as albumin synthesis, urea production, and cytochrome P450 activity, reflecting that of a native liver in vitro [28]. Furthermore, Ott and his team developed lung scaffolds featuring acellular vasculature, airways, and alveoli from decellularized pulmonary tissue, verifying functional performance in a bioreactor setting prior to transplantation. In 2013, Song et al. applied the decellularization technique to rat, porcine, and human renal tissues, producing acellular scaffolds that encompassed vascular, cortical, and medullary architectures, along with the collecting systems and ureters. These recellularized renal scaffolds were assessed for host integration and functionality in vivo, with urine output observed using an arterially perfused ex vivo bioreactor system [29]. In the same year, Chan et al. demonstrated that traditional decellularization approaches for thin tissues could be effectively applied to sizable organs, such as the intervertebral disc (IVD). The decellularized IVDs preserved essential components, including glycosaminoglycan concentrations, collagen fiber architecture, and mechanical integrity, thereby indicating promising avenues for IVD bioengineering and the development of cell culture scaffolds [30].

In 2016, Zhang and colleagues progressed the engineering of vascularized soft tissue constructs by combining decellularized skin/adipose tissue matrices with a variety of human cell types. This innovation enabled the effective regeneration of large-scale adipose tissue in vivo, establishing the groundwork for the development of composite soft tissue constructs in the realm of microsurgical reconstruction [31]. Similarly, ECM scaffolds derived from porcine sources have shown promise as supportive microenvironments favorable to the regeneration of adipose tissue [32]. Turksen et al., in 2017, showed the practicality of combining decellularized cartilaginous matrices from donor tracheae with diverse hydrogels, thereby forming scaffolds suitable for cartilage tissue engineering [33].

The scope of studies focusing on hydrogel applications using decellularized scaffolds has broadened considerably, with ECM hydrogels derived from decellularized tissues currently utilized in various clinical settings to enhance tissue structure and promote functional remodeling. In 2018, Gaetani and colleagues evaluated the impact of diverse decellularization protocols on the mechanical properties and biochemical composition of pancreatic ECM hydrogels, noting an increased collagen content compared to that of fresh pancreas tissue [34]. Concurrently, Lin and team demonstrated that decellularized porcine neural matrices supported Schwann cell proliferation and peripheral nerve regeneration, owing to the preservation of primary ECM components and nanofiber architectures. This finding has prompted additional investigations into decellularized peripheral nerve matrix hydrogels for the repair of nerve defects [35]. In 2019, Giobbe et al. verified that hydrogels sourced from decellularized porcine small intestinal mucosa/submucosa encouraged the formation and growth of endoderm-derived organoids, including those from the stomach, liver, pancreas, and small intestine [36].

With advances in 3D bioprinting technology, Pati et al., in 2014, formulated a technique for bioprinting cell-laden constructs using innovative dECM bio-inks, establishing an optimized microenvironment favorable to three-dimensional tissue development. They emphasized the adaptability and versatility of the bioprinting methodology by using tissue-specific dECM bio-inks for adipose, cartilage, and cardiac tissues. This approach delivered crucial signals essential for cell implantation, viability, and sustained functionality [37]. In 2017, Jang and colleagues utilized dECM bio-inks enriched with stem cells for the 3D bioprinting of prevascularized and functional multimaterial constructs [38].

Despite the abundance of studies centered on decellularized scaffolds, a robust and comprehensive characterization of the human decellularized scaffold proteome remains elusive. Li’s pioneering investigation in 2016 marked the first report of a proteomic assessment of partially functional decellularized organs, clarifying specific protein losses incurred during the decellularization process. These insights have enhanced our understanding of biological scaffolds and highlighted the necessity of integrating proteomics into tissue engineering research [39]. In 2021, a study led by Amish Asthana et al. introduced an enhanced workflow for comprehensive proteomic analysis that combines mass spectrometry and multiplexed ELISA techniques, establishing a benchmark for probing the proteome of human decellularized scaffolds [40]. This review will strive to comprehensively characterize the ECM proteome and provide guidance to refine the design and engineering strategies of tissue engineering scaffolds.

### Major components of extracellular matrix

**Fig. 2:**
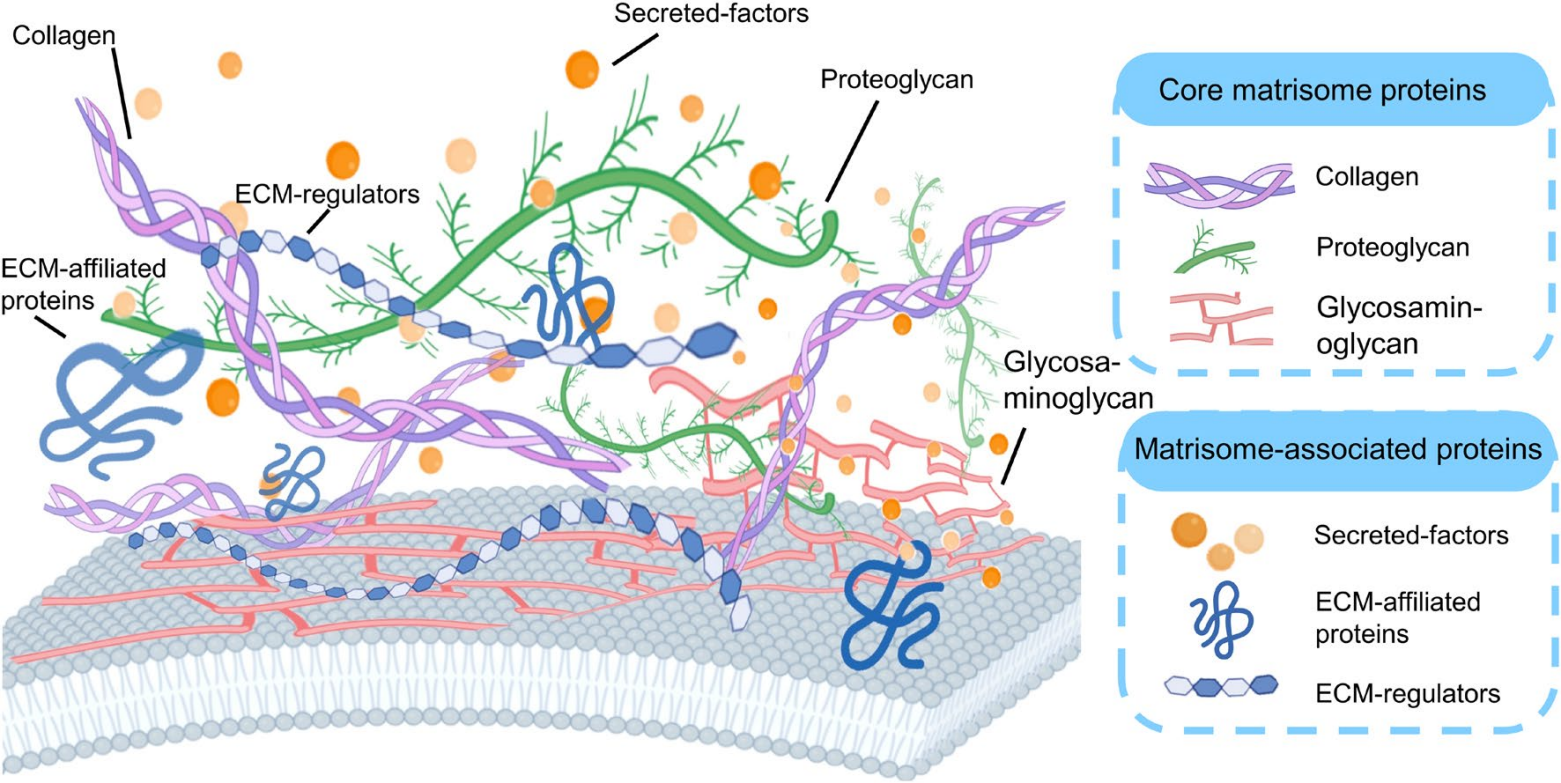
The main components of ECM: collagen, proteoglycans, glycoproteins, ECM-related proteins, ECM regulators and secretory factors

The ECM primarily comprises structural and functional macromolecules such as collagens, proteoglycans, and glycoproteins, augmented by cytokines and other bioactive molecules (see Fig. 2). This intricate and dynamic network of proteins plays a vital regulatory role in cellular processes including proliferation, survival, differentiation, and migration. With the introduction of the Matrisome concept, Hynes and Naba in 2012 introduced a comprehensive tool for analyzing the composition of the ECM in both normal and pathological tissues, leveraging extensive mass spectrometry data from various studies [42]. The development of the Matrisome database has undergone several significant phases. Initially, it included proteomic data for ECM from 17 studies encompassing 15 types of normal tissues, six cancer types, and other diseases, including vascular anomalies and pulmonary and hepatic fibrosis. With the release of the Matrisome database 2.0, the database was substantially expanded to include data from 25 new studies on 24 additional tissue types of ECM, along with more comprehensive data sets for previously included tissues, achieving near-complete coverage of predicted matrisome proteins [41]. The Matrisome database categorizes ECM proteins into two primary groups: core matrisome proteins and matrisome-associated proteins. The core matrisome proteins in humans comprise nearly 300 proteins, including 45 collagen subunits, 36 proteoglycans, and 168 glycoproteins. Matrisome-associated proteins are further classified into ECM-affiliated proteins, ECM regulators, and secreted factors.

Collagen, the most abundant protein in the ECM, forms heterotrimeric alpha chain helices, which can assemble into either homotrimers or heterotrimers. It is present in various forms within mammalian tissues, with types I, II, III, IV, V, and VI being the most common [43, 44]. Types I, III, and V often coexist, with type I being prevalent in connective tissues due to its strong tensile strength, which facilitates the formation of a fibrous network in conjunction with elastin and keratin. This network enhances the resistance of tissues and organs to mechanical forces. Type II collagen, primarily located in hyaline cartilage and the nucleus pulposus, has functions that remain largely unclear but is associated with connective tissue disorders. Type III collagen, found in skin, lungs, and blood vessels, is often observed alongside type I [45]. Type IV collagen forms the basement membrane, offering mechanical support and tensile strength through covalent bonds [46, 47]. Type V collagen, which coexists with type I, can regulate the assembly of heterotypic fibers, while mutations in type VI collagen, a component of muscle microfibrils, lead to Bethlem myopathy.

Proteoglycans, interspersed among collagen fibers within various ECMs, are composed of glycosaminoglycans (GAGs). These are glycoproteins featuring carboxylic and sulfuric acid groups. A diverse array of GAGs, including heparin, heparan sulfate, chondroitin sulfate, and hyaluronic acid, is present on the cell surface and within the ECM. These GAGs boast the capacity to interact with a multitude of growth factors and secreted molecules [48–50]. Additionally, owing to their exceptional hydration properties, GAGs predominantly inhabit intercellular spaces [51].

Beyond collagens and proteoglycans, which provide strength and volume, the mammalian matrisome includes approximately 200 complex glycoproteins. These glycoproteins play a crucial role in facilitating ECM assembly, promoting cell adhesion through specific domains and motifs, cellular signaling, and binding growth factors, which can either act as reservoirs for release or function as solidphase ligands through ECM proteins. Among the most extensively studied ECM glycoproteins are laminins and fibronectins [52–54]. Other notably characterized glycoproteins include thrombospondins and tenascins [52, 55]. These glycoproteins display multiple repeating domains and extended multimeric forms, typical of ECM proteins, a characteristic also found in fibulins, nidogens, and others [56]. Moreover, ECM glycoproteins, such as fibrillins, have been studied in disease contexts and in the regulation of TGF-β functions [57].

The ECM microenvironment consists of both insoluble and soluble components. As detailed in the existing literature [48, 49, 58–61], a variety of growth factors interact with ECM proteins, which are crucial constituents of the ECM. A widely accepted hypothesis suggests that growth and other secreted factors primarily bind to GAGs, particularly heparan sulfates, although they also bind to specific domains of ECM proteins. For example, fibronectin distinctively interacts with several growth factors such as VEGF, HGF, and PDGF [54, 62–64], and domains such as VWC/chordin and follistatin in ECM proteins specifically bind to BMPs [2, 65]. The ECM serves as a reservoir for such factors, influencing developmental signals and creating gradients that direct pattern formation, which are significantly altered by ECM interactions.

### Obtaining the decellularized extracellular matrix

The optimal preservation of ECM components requires meticulous management of decellularization techniques. The conceptualization of ECM and the refinement of decellularization methods have undergone extensive research. This section offers a comprehensive review of the current state of decellularization technology, detailing the predominant methodologies.

#### Decellularization methods

**Fig. 3:**
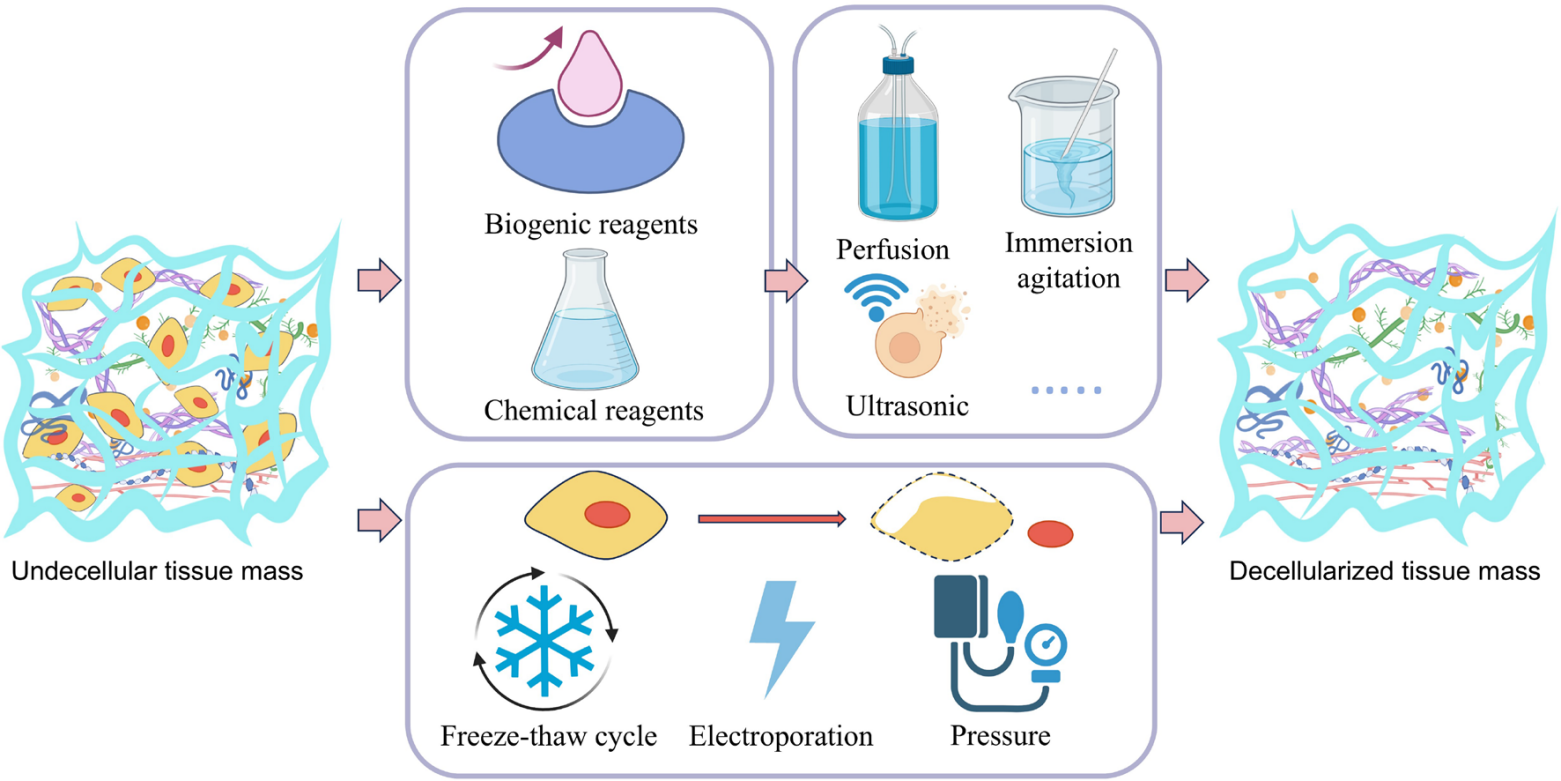
Transitioning from tissue to ECM requires decellularization, a process which falls into two broad categories. The first involves the selection of a reagent—either biological or chemical—followed by the application of decellularization methods such as perfusion, immersion stirring, or ultrasound. The second primarily employs physical means to disrupt the cell membrane for decellularization, with methods including freeze–thaw cycles, electroporation, and pressure

Decellularization technology commonly comprises two fundamental components: the selection of decellularization reagents and the tissue processing methods, as shown in Fig. 3. Decellularization techniques are meticulously designed to preserve the ECM’s bioactivity, which is essential for organoid integration and function. The selection of decellularization reagents and the optimization of tissue processing methods are critical for maintaining the ECM’s structural integrity and its bioactive components, such as growth factors and cytokines, which play pivotal roles in cellular processes like adhesion, proliferation, and differentiation.

Decellularization reagents are categorized into two primary types: chemical reagents and biogenic reagents, with the latter primarily comprising enzymes and chelating agents. Chemical reagents primarily encompass ionic and non-ionic surfactants, acid–base solutions, hyperosmotic solutions, and organic compounds. Prominent examples of surfactants comprise sodium dodecyl sulfate (SDS), sodium deoxycholate (SDC), sodium dodecyl glutamate, sodium dodecyl ester sulfate, TritonX-100, and TritonX-200 [66]. Their primary mechanism of action entails the dissolution of the plasma membrane, nucleic acid membranes, nucleic acids, and the separation of DNA from proteins. Nonionic detergents exhibit a gentler impact than ionic detergents and are ideally suited for preserving the structure and nutrients of the ECM; however, their process is time consuming, and eliminating residual substances within the ECM proves challenging.

The employment of potent acids and bases is a frequent practice in the decellularization process, incorporating agents such as peracetic acid, hydrochloric acid, ammonium hydroxide, sodium hydroxide, and sodium sulfide. These reagents operate by disrupting the cellular membrane, liquefying the cytoplasm and organelles, inducing cell lysis, denaturing proteins, and accelerating the hydrolysis and decomposition of biological molecules, thereby facilitating efficient cell removal [67–69]. Given the robust nature of these acids and bases, glycosaminoglycans (GAGs) and associated growth factors are prone to rapid degradation, and cellular remnants are effectively eradicated. However, it is important to emphasize that these stringent conditions may also eliminate a portion of bacterial contaminants. Moreover, it is imperative to recognize that prolonged exposure to acidic or alkaline environments may result in structural modifications in the extracellular matrix (ECM), potentially causing damage to critical components such as collagen fibers and fibronectin.

Within the context of cellular environments, normal cells generally sustain a dynamic equilibrium of osmotic pressure. Disruptions in this balance, induced by hypertonic or hypotonic solutions, can lead to significant water loss or uptake by cells, respectively. This osmotic imbalance can culminate in the destruction of cellular structures and eventual cell death once certain thresholds are exceeded, thereby expediting the process of decellularization [67]. While this method typically preserves the ultrastructure of the ECM, it does not consistently achieve complete DNA removal and frequently requires prolonged treatment durations. Commonly, a hypertonic solution with a high concentration of sodium chloride is utilized, whereas tris(hydroxymethyl)aminomethane hydrochloride functions as the hypotonic solution. Additionally, organic solvents such as methanol, ethanol, and acetone are employed to induce cell death by compromising cellular membrane integrity and increasing membrane permeability. Moreover, some organic solvents demonstrate strong hygroscopic characteristics, resulting in cellular dehydration similar to the effects observed with hypertonic solutions.

The final major category of decellularization agents encompasses substances of biological origin, primarily enzymes, with chelating agents also contributing significantly. Given that enzymes function by hydrolyzing specific sites, they lack the specificity to distinguish between sites within the ECM that should remain intact, which could significantly impact the ECM’s structure and protein composition. For instance, trypsin can undermine the structural integrity of elastin and collagen, while dispase is known to target and cleave specific peptides, such as collagen type IV and fibronectin in the basement membrane, thereby affecting the end products [66, 70]. As a result, the concentration and duration of enzyme exposure must be carefully regulated to optimize decellularization while maximizing the preservation of the ECM’s integrity. Chelators such as EDTA and EGTA promote cell detachment from the ECM by chelating divalent metal ions essential for cell adhesion, a process that could potentially disrupt protein–protein interactions and denature proteins within the ECM.

The choice of decellularization agents is crucial for retaining the ECM’s bioactivity. For instance, the use of nonionic detergents and mild enzymatic treatments is favored for their ability to gently remove cellular components while preserving the ECM’s protein structure and bioactive molecules. This careful balance ensures that the resulting dECM scaffolds support organoid attachment, growth, and differentiation, akin to their in vivo counterparts.

Decellularization reagents are typically employed in conjunction with methods such as perfusion, immersion agitation, acoustic degradation, and the use of supercritical fluids [3, 66, 68, 71]. Through the combined application of these techniques, decellularization reagents can more effectively permeate tissues, significantly enhancing the rate of decellularization. Perfusion is commonly utilized for highly vascularized tissues, such as the liver and kidney perfusable [28, 72, 73]. The decellularization agent acts within the vascular system of the tissue or organ, enabling a more uniform and expedited process. This approach not only removes cellular components but also clears vascular-related elements from the ECM, albeit necessitating a comprehensive perfusion system and proficient operation. Immersion and agitation are generally suitable for a broad range of tissues, irrespective of their vascularization. However, immersion does not provide the same depth of penetration as perfusion, resulting in limited decellularization efficiency. Nonetheless, this method minimizes tissue deformation and preserves the microstructure, collagen framework, and overall integrity of the decellularized scaffold. Stirring following immersion in the decellularization reagent can enhance efficiency and reduce the duration of decellularization, thereby minimizing damage to the ECM’s structure and components. Acoustic degradation uses vibrational energy to generate cavitation bubbles, which disrupt cell membranes and assist in the penetration of decellularization reagents and the removal of cellular debris [74]. Supercritical fluid technology involves a phase in which the liquid state has no clear gas–liquid interface [75]. In this state, decellularization reagents exhibit enhanced penetration efficiency and improved decellularization efficacy. However, the number of decellularization reagents capable of reaching a supercritical state is limited, thus constraining the application of this method.

In addition to the aforementioned methodologies, the dECM can also be obtained using a single treatment approach. The most basic amongst these is the freeze–thaw cycle, which utilizes intracellular water to form ice crystals upon freezing, consequently rupturing the cell membrane; subsequent thawing permits the release of cellular contents. However, this technique can potentially damage the microstructure of the ECM, and residual fragments of the cell membrane may adversely affect the ECM components. Other single-treatment methods that disrupt the cell membrane include the application of pressure and electroporation, both of which accelerate cell lysis [76].

Nevertheless, the implementation of a multifaceted approach involving both de-cellularization reagents and methodologies has emerged as a modern trend. The combined use of chemical and biological agents can significantly enhance the rate of decellularization and improve the quality of the resulting decellularized scaffolds [76, 77]. Shafiq et al. utilized a combination of SDC, freeze–thaw cycles, nuclease, and trypsin to address the challenges associated with residual tissue following prolonged exposure to chemical reagents, and to tackle the degradation of various structural proteins in the ECM due to extended enzyme application [78].

#### Criteria for the degree of decellularization

Currently, the field lacks a universally accepted set of criteria for assessing the effectiveness of decellularization in tissues or organs, which hinders progress in the realm of ECM applications. Decellularization endeavors typically pursue two principal goals: the complete removal of cellular constituents and the careful preservation of ECM components. The challenges of immunogenicity, ECM microstructural alterations, and the residual presence of cellular materials are inherent to the decellularization process. As a result, numerous investigative teams rigorously assess ECM decellularization quality across four critical dimensions: compositional analysis, quantification of cellular remnants, evaluation of residual decellularization agents, and assessment of structural integrity.

The ECM’s fundamental composition includes collagen, proteoglycans, elastin, laminin, fibronectin, and GAGs. Changes in tissue composition before and after decellularization can be measured through techniques such as immunohistochemistry, immunofluorescence staining, and Western blot analysis. Given that DNA and genetic materials are hallmarks of cellular remnants, Crapo and colleagues have delineated minimum standards for assessing the removal of such residues, stipulating that decellularized ECM should harbor less than 50 ng of DNA per milligram of dry ECM, DNA fragments should not exceed 200 nucleotides in length, and histological sections stained with DAPI or hematoxylin and eosin should exhibit no visible genetic material [79].

The detection of residual decellularization agents is crucial for determining ECM quality. The substantial reduction of chemical residues significantly improves the ECM’s overall integrity. Furthermore, the mechanical properties of the ECM serve as indicators to measure the impact of decellularization, employing metrics such as tensile strength, elastic modulus, viscoelastic properties, stiffness, and yield strength to provide benchmarks for assessing the thoroughness and quality of decellularization. The establishment of precise criteria for ECM evaluation requires more extensive standardization in the field.

## Sterilization and preservation of extracellular matrix after acquisition

### Sterilization methods

Prior to the application of decellularized biomaterial scaffolds, sterilization and the removal of residual genetic material, bacteria, and viruses are essential to minimize the risk of immunogenicity. Common terminal sterilization techniques include gamma irradiation, electron beam irradiation, ethylene oxide, and supercritical carbon dioxide. Antibiotics and antifungals are routinely used for sterilization during decellularization and aseptic processing [80]. Furthermore, the chosen sterilization method must be suitable for the size and complexity of the decellularized tissue graft and avoid structural damage and ECM alteration. Sterilization methods are divided into physical, chemical, and biological classifications (Table 1).

**Table 1:** List of sterilization methods for preparing dECM scaffold

Sorts	Method	Mechanism	Advantage	Disadvantage	Applicability	References
Physical sterilization	High temperature and high-pressure sterilization	Disrupt the biomolecular structure of microorganisms and alter the physical and chemical properties of substances	Effective eradication of broad-spectrum microorganisms with minimal impact on the physical and chemical characteristics of the scaffold	The irreversible change of thermosensitive materials affects the bioactivity of the scaffold	Bone scaffolds and arterial vessels	[81, 82]
	Radiation sterilization	Direct disruption of nucleic acids, proteins, and enzymes in microorganisms through its potent penetration capability	No significant temperature elevation by disinfectant products	Changes in the physical and chemical properties, as well as biocompatibility of dECM	High biological activity scaffold material	[83, 84]
	Supercritical carbon dioxide (scCO2)	Disruption of microbial cell membrane structure	Negligible impact on structural integrity and mechanical performance of transplants and relatively non-toxic	Extensive research required to ascertain its disinfection efficacy without compromising the substrate	Natural and synthetic biomaterials	[85, 86]
Chemical sterilization	Etherification, ketonization, and alcoholization	Modes of action utilizing chemical agents: oxidative reductive reactions and protein crosslinking	Effective against broad spectrum microorganisms with fast and low-cost characteristics	Potential toxicity and residue production may exist	Thermally sensitive scaffold materials	[87, 88]
Biological sterilization	Antibiotics and antifungal agents	Inhibiting protein and DNA synthesis by disrupting bacterial cell wall	Maximizing reduction of microbial contamination during decellularization process	Ineffective against viruses and spores, limited antibacterial spectrum	Kidney tissue and cardiovascular scaffolds	[84, 86, 89]

#### Physical sterilization

Physical sterilization encompasses methods such as autoclaving (high-temperature), high-pressure, and irradiation techniques [82]. The paramount advantage of physical sterilization lies in its omission of chemical reagents, consequently mitigating potential environmental pollution and lessening the risk of scaffold toxicity. Furthermore, these methodologies generally exhibit efficacy in eradicating microbial contaminants. However, physical sterilization frequently necessitates specialized equipment, incurs substantial costs, and possesses the potential to alter the structural integrity of scaffolds. Autoclaving, proficient in eliminating a broad spectrum of microorganisms, impinges minimally on the physical and chemical attributes of the scaffold, albeit it may irreversibly denature thermosensitive materials. This method is predominantly employed for bone scaffolds and decellularized arterial vessels. High-pressure sterilization, which utilizes pressure to exterminate microbes, guarantees sterility in scaffolds. While this technique efficaciously neutralizes the majority of microbial life, it may impact the bioactivity of the scaffold.

Radiation sterilization harnesses the power of ionizing radiation, which includes gamma rays and electron beams, to eradicate microbial contaminants within scaffolds. Gamma irradiation (GI) is acknowledged as a non-thermal process that maintains the ambient temperature of the sterilized materials, thus preserving their suitability for the sterilization of biomaterials. This method utilizes the radioactive isotope cobalt-60 as its radiation source. Notably, products subjected to cobalt-60 gamma radiation do not retain radioactivity due to the insufficiency of energy to induce such a state, yet this modality efficiently obliterates extant microbes [80, 90]. In parallel, electron irradiation (EI) represents an alternative non-thermal sterilization technique, employing electron accelerators to generate the requisite radiation [83, 91]. Both GI and EI mechanisms are recognized to inflict damage upon DNA, thereby thwarting microbial proliferation within grafts; however, they may concurrently compromise the integrity of the ECM network. It has been reported that low-dose irradiation can promote cross-linking within dECM scaffolds, whereas high-dose irradiation may lead to ECM denaturation and degradation. Such alterations can result in reduced mechanical robustness and diminished capacity for cell adhesion. Studies have indicated that gamma irradiation at 5 kGy is particularly deleterious, undermining mechanical properties, distorting the microstructure of the kidney matrix, and impeding cellular adhesion. Additionally, ultraviolet irradiation has been shown to be ineffectual in the sterilization of decellularized tissues.

Supercritical carbon dioxide (scCO2) is recognized for its exceptional sterilant properties and is utilized in the decontamination of a diverse array of biomaterials, both of natural and synthetic origin [86]. By virtue of its reduced viscosity and enhanced diffusion coefficient, scCO2 can permeate biological scaffolds, effectively eliminating undesirable substances while preserving the essential structural integrity and biomechanical characteristics of the grafts. This attribute is particularly advantageous for dense tissue matrices, where comprehensive penetration is imperative for the successful execution of decellularization and sterilization protocols. Moreover, the relative non-toxicity of scCO2 establishes it as a preferable technique for the decellularization and sterilization processes in the fabrication of tissue-engineered constructs that are devoid of immunogenicity [85, 86].

#### Chemical sterilization

Numerous chemical sterilization methodologies, such as etherification, ketonization, and alcoholization, constitute a critical component of the arsenal against microbial contamination. The efficacy of these chemical approaches is widely acknowledged due to their ability to neutralize a wide spectrum of microorganisms, encompassing both bacteria and fungi, and their rapid and economically viable nature. Nonetheless, potential drawbacks such as the toxicity or residual presence of certain chemical agents within scaffold materials necessitate comprehensive safety evaluations and additional decontamination efforts.

Chemical sterilization is particularly applicable to thermosensitive scaffold materials, notably including decellularized cartilage scaffolds [92]. Although 70% ethanol exhibits the capability to denature proteins, its inability to eradicate bacterial spores compromises its suitability for the disinfection of dECM scaffolds. On the other hand, sterilization with ethylene oxide (EtO) involves exposing materials to EtO gas. Acting as an alkylating agent, EtO obstructs DNA functionality, thereby curtailing microbial replication, as well as cellular metabolism and division. However, the penetration depth of EtO is relatively limited, affecting mainly the material’s surface layers. Furthermore, the intrinsic toxicity and carcinogenic properties of EtO have raised significant concerns; it is known for leaving behind immunogenic residues that potentially impair the biocompatibility of scaffolds. Consequently, despite its sterilizing efficacy, the use of EtO is increasingly regarded with caution and considered a less preferable option for the effective sterilization of biomedical scaffolds, as indicated by the literature [87, 88].

#### Biological sterilization

During the decellularization process, the use of antimicrobial agents, such as penicillin, streptomycin, amphotericin B, and sodium azide, has been instrumental in curtailing microbial contamination [93, 94]. Empirical studies have shown that sterilization with 0.5% peracetic acid (PAA), combined with an antibiotic cocktail, effectively sterilizes biomaterial scaffolds. This method not only preserves the mechanical integrity of the scaffolds but also retains the critical components essential for facilitating cell–matrix interactions and cellular adhesion. This has been especially evidenced in the thorough purification of decellularized rabbit kidney tissues [84, 95]. Nonetheless, given its narrow spectrum of pathogen eradication, PAA sterilization is best utilized as an ancillary measure alongside other sterilization strategies.

Research focused on optimizing sterilization protocols for porcine and bovine decellularized pericardium has shown that an initial treatment with antibiotic and antifungal agents, followed by PAA processing, effectively preserves the tissue’s structural and compositional fidelity [89]. Furthermore, this sequential approach guarantees the retention of biocompatibility and biomechanical properties, thus enhancing the clinical applicability of the sterilized tissues.

### Preservation of dECM scaffolds

Considering the constraints on immediate availability, the use of fresh decellularized tissue constructs often is impractical, making long-term preservation techniques essential. The primary objective of preservation is to ensure the readiness of clinically applicable products. Therefore, the development of suitable preservation strategies is crucial in maintaining the quality and enabling the clinical translation of these tissue-derived products.

Standard preservation methods include lyophilization, cryopreservation, and storage at sub-zero temperatures such as − 20 °C or − 80 °C, usually combined with antibiotics and antifungal agents (see Table 2). The careful selection of a preservation method is essential to maintain the structural integrity and sustain the biological activity of decellularized scaffolds.

**Table 2:** List of dECM scaffolds preservation methods

Method	Mechanism	Advantage	Disadvantage	Applicability	References
Cryopreservation	Use 10% DMSO and rapid freezing in liquid nitrogen	Retain structure and regenerative potential, enable an extended shelf life	Tissue architecture and biomechanical properties degradation, clinical applicability limitation	Most tissues and organs	[96–98]
Lyophilization	Water removal by vacuum sublimation process	Maintain tissues integrity and biological activity	Require subsequent reconstitution step	Skin and pulmonary valve	[83, 99]
Immersion preservation	Store scaffolds in specific liquids such as antibiotics, ethanol, mannitol, saline, or preservative-containing solutions	Maintain the scaffold’s hydrated state, preserve biological activity	Prolonged immersion may alter the scaffold’s structure, and preservatives may be cytotoxic	Pulmonary tissue	[68, 100]

#### Cryopreservation

Cryopreservation entails the use of 10% dimethyl sulfoxide (DMSO) combined with either controlled-rate freezing or rapid immersion in liquid nitrogen. This method is essential for preserving the histological integrity of tissue grafts at levels comparable to those of fresh grafts [98]. Additionally, other long-term preservation strategies involve storing decellularized tissue constructs in phosphate-buffered saline supplemented with antibiotics and antifungal agents, at 4 °C for short-term storage, and at − 20 °C or − 80 °C for longer periods [101]. Despite these advancements, current preservation methods result in a gradual deterioration in tissue architecture and biomechanical properties. This degradation restricts the clinical applicability of preserved organs and emphasizes the urgent need for continued research in this area to enhance preservation outcomes.

While cryopreservation may cause ice formation and resulting damage to the extracellular matrix (ECM), vitrification avoids ice crystallization during the cooling and warming processes, thereby emerging as a superior method for tissue and organ preservation. This technique is widely used for the long-term storage of viable cells and tissues. Narine and colleagues assessed the viability of cryopreserving porcine aortic valve matrices and found that while cryopreservation did not significantly alter the biochemical properties of the matrices, it adversely affected their structural and mechanical attributes [97]. Theodoridis et al. conducted a comparative analysis of the biomechanical characteristics, structure, and biochemical properties of decellularized porcine pulmonary valves before and after cryopreservation, concluding that scaffolds cryopreserved after decellularization exhibited better outcomes compared to those decellularized after cryopreservation [102].

The use of cryoprotectants during cryopreservation can reduce ice crystal formation, thereby improving scaffold preservation. Feng et al. demonstrated the effective cryopreservation of decellularized kidney scaffolds by incorporating high concentrations of the cryoprotectant VS83, with computed tomography (CT) results confirming the preservation of vascular networks and tissue architecture [103]. Concurrently, Brockbank and colleagues suggested that heart valves, preserved with VS83, could be maintained and transported at temperatures close to − 80 °C, thereby preserving ECM integrity and material properties [104]. Therefore, the impact of cryopreservation on tissue organs is relatively minimal; cryopreserved decellularized organs maintain their structure and regenerative potential, thereby extending their shelf life and enhancing their clinical application.

#### Lyophilization

To preserve decellularized tissue grafts over extended periods, lyophilization (freeze–drying) is utilized to maintain material integrity without causing substantial structural damage during processing. Reduced pressure is applied to prevent the formation of large ice crystals that might physically damage the tissues, and during sublimation, most water in the material transitions directly from solid to gas. Following this phase, the temperature is increased to disrupt the hydrogen bonds between the water molecules and the ions within the construct, thereby further drying the construct. This method employs non-toxic cryoprotectants and eliminates the need for low-temperature storage [105]. Goecke et al. assessed lyophilized decellularized heart valves using trehalose as a cryoprotectant and determined that lyophilization does not compromise the early hemodynamic performance or recellularization potential of decellularized grafts in juvenile sheep, retaining satisfactory early function [83]. Sun et al. documented the use of trehalose and DMSO for lyophilizing 3D tissue-engineered skin grafts, evaluating their regenerative effect on murine skin defects [99]. In vivo studies showed that lyophilized tissue-engineered skin facilitated effective healing of skin defects after 4 weeks of storage.

#### Immersion preservation

Immersion preservation involves the storage of scaffolds in specific liquids such as ethanol, mannitol, saline, or preservative-containing solutions. The advantage of this method is its ability to maintain the scaffold’s hydration, beneficial for preserving its biological activity. However, prolonged immersion may compromise the scaffold’s structure, and preservatives may be cytotoxic. Ethanol, which is effective at killing most bacteria and viruses, is commonly used for preserving decellularized scaffolds, but it can impair certain biological activities and requires thorough washing before use to remove residual ethanol [68]. Mannitol, a high-concentration sugar solution, inhibits ice crystal formation in the cellular matrix during freezing, thereby protecting the matrix’s integrity and function. This method is frequently employed for cryopreserving decellularized scaffolds, although it necessitates washing to eliminate residual mannitol before use [100]. Saline effectively reduces structural disruption of the cellular matrix, preserves scaffold integrity, and is typically used for short-term storage; however, extended storage in saline can result in degradation of the scaffold [68].

## Promising use of extracellular matrix: preparation of hydrogels

In the exploration of pragmatic implementations of the extracellular matrix (ECM), the primary focus of researchers centers on two cardinal attributes: structural reinforcement and bioactive molecule conveyance. From a structural standpoint, the predominant methodology involves the use of biocompatible polymers such as gelatin, hyaluronic acid, chitosan, among others, to effectively mimic the ECM and provide a mechanical scaffold reminiscent of its natural counterpart [106]. As for the examination of bioactive compounds within the ECM, a bidimensional research model is frequently adopted, whereby the compound of interest is integrated into the culture medium [107]. These dualistic approaches towards understanding the functionality of the ECM have established a solid research foundation, the results of which have been partially employed and innovated. However, achieving simultaneous exploitation and execution of the ECM’s dual characteristics remains an ongoing challenge. As a result, efforts are being directed towards optimizing the ECM’s functionality to broaden its range of applications.

### Improvements in organoid culture

The 3D architecture of the ECM has attracted considerable attention in the context of organoid research in recent years, as it not only provides structural support but also orchestrates the complex cellular interactions that drive tissue-specific organization and function. This 3D environment is critical for the maintenance of cellular phenotypes and the directional cues necessary for proper tissue morphogenesis [108, 109].

The maturity of organoids is significantly influenced by the 3D context in which they are cultured. ECM hydrogels provide the necessary spatial cues and mechanical support that promote the differentiation of stem cells into functional tissue-specific cells, enhancing the functional maturity of organoids and their ability to recapitulate in vivo tissue functions.

Matrigel and basement membrane extract (BME) are the most commonly used media for organoid culture; however, they contain tumor-derived substances, exhibit inter-batch variability, and are murine-derived, potentially impacting experimental results and, to some extent, limiting the development and application of organoids [11]. Therefore, integrating the ECM with organoid culture has the potential to transform the ECM or its essential components into a hydrogel akin to an adhesive matrix, thus facilitating and enhancing the application and development of organoids. Commercially available matrix gels are thermosensitive hydrogels that transition from a liquid to a gel at temperatures between 22 and 35 °C when pre-cooled. Pre-cooling all experimental apparatus is necessary during the experimental procedure. These gels primarily consist of proteins, polysaccharides, and synthetic polymers. While these commercial gels have inherent limitations, their composition is simpler compared to the ECM.

### An overview of ECM hydrogels

ECM hydrogels have been extensively suggested as analogues of the biological milieu encountered by cells within native tissues [110–113]. Owing to their 3D structure and hydrophilic nature, these hydrogels can retain substantial quantities of water or biological fluids, thus closely simulating the in vivo environment. Presently, independently sourced extracellular matrices exhibit complex compositions with variable content levels of principal constituents, due to intrinsic disparities and state variations in tissues and organs, thus posing considerable challenges in gel formation.

Consequently, the incorporation of ECM components into these hydrogels appears highly promising. A well-established strategy for hydrogel preparation involves lyophilizing the acquired ECM, converting it into a powder, and subsequently dissolving it in an acidic solution with pepsin [3]. Following enzymatic hydrolysis, the insoluble material is eliminated, the pH is adjusted, culminating in the critical step of crosslinking. ECM hydrolysates can spontaneously assemble and reorganize into gels; however, their innate crosslinking properties are closely associated with the enzymatic state of ECM’s main components and the external conditions for gel formation.

Hydrogels can be engineered and synthesized through chemical (e.g., free radical polymerization, addition reactions, REDOX reactions) and physical (e.g., ionic interactions, hydrogen bonding, crystallization) crosslinking techniques. Furthermore, molecules with photocrosslinkable properties can be grafted onto the native ECM, or photocrosslinkable substances can be incorporated. Gelatin methacryloyl (GelMA) is a widely-used photocrosslinkable hydrogel derived from collagen, a natural biological material. The methacrylate group, grafted onto the gelatin structure, triggers free radical polymerization under ultraviolet irradiation, resulting in a covalently crosslinked hydrogel.

## Characteristics of ECM hydrogels

Irrespective of the various applications of extracellular matrix hydrogels—ranging from cell culture and organoid development to wound management and tissue restoration—the critical evaluation criteria extend beyond the fundamental bio-compatibility and biodegradability to encompass the adaptable topological structure, mechanical attributes, and microstructure. Numerous properties of hydrogels in this context derive from the auxiliary materials or proteins that possess deformability, collectively referred to as their mechanical characteristics. Nonetheless, the primary constituents of the extracellular matrix also include bioactive substances such as proteoglycans and cytokines. The concentration of these substances within hydrogels can be precisely controlled, thereby determining the biochemical traits of hydrogels.

In essence, the extracellular matrix influences cells in the following ways: by offering mechanical signals to cells through substrates of varying stiffness; by regulating the accessibility and activity of soluble factors; and by triggering intracellular signaling via cell adhesion molecules [99]. Consequently, hydrogels constructed from the extracellular matrix embody these functionalities as well. In the subsequent sections, hydrogels derived from the extracellular matrix will be examined from various perspectives, and based on this analysis, strategies for modifying the hydrogels will be proposed.

### Biocompatibility and biodegradability

Biocompatibility is an essential feature of hydrogels and a prerequisite for their diverse applications. Biocompatibility requires that the material does not impair cellular and tissue function upon contact with living tissues and bodily fluids, nor does it induce inflammation, carcinogenesis, or rejection. During the acquisition of the extracellular matrix, residual immunogenic substances, such as decellularization agents and non-decellularized cellular components, might persist. Experimental methods for evaluating biocompatibility include cytotoxicity testing, genotoxicity and carcinogenicity assays, dominant lethal testing, implantation studies (subcutaneous, intraosseous), and allergy testing, among others. Practically, for assessing the biocompatibility of hydrogels, beyond cytotoxicity kit testing, it is customary to implant hydrogels subcutaneously in mice. After a designated period, the surrounding tissues are excised for histological analysis (H&E and toluidine blue staining) to evaluate tissue morphology, damage, and inflammatory cytokine accumulation.

Biodegradability is crucial in the vascularization process of organoids. One objective of organoid development is the in vivo transplantation of functional organoids, with the aim of extending the wait time for organ transplants or ameliorating or reversing functional deterioration due to organ pathologies. Therefore, biodegradability constitutes a crucial characteristic for organoids intended for in vivo implantation. Biodegradation refers to the progressive elimination of materials from the body via dissolution, enzymatic hydrolysis, cellular phagocytosis, and other processes concurrent with tissue development within the organism. After repair, the regenerated tissue entirely supplants the site of the implanted materials, leaving no residual material within the body. Currently, prevalent biodegradable materials include peptides, hyaluronic acid (HA), polyamino acids, polyesters, polylactic acid, chitin, bone collagen, gelatin, and others [114]. These natural hydrogels are readily enriched, demonstrate favorable biocompatibility, and are amenable to modulation in response to external stimuli, such as temperature, pH, and ionic conditions. However, they also exhibit limitations, including low stability, suboptimal mechanical properties, and rapid degradation. The compatibility of various tissues with distinct types of biodegradable materials varies significantly. For instance, Hunt demonstrated that RGD-alginate-saline gels outperformed HA-based hydrogels in delivering retinal cells to the damaged retina [115].

### Mechanical properties of hydrogels

Hydrogels derived from the extracellular matrix (ECM) are essential in emulating the mechanical properties of diverse tissues and organs, which play a crucial role in determining cell fate and behavior. The topological architecture and mechanical integrity of ECM hydrogels are crucial for stability and significantly influence organoid differentiation and functionality. Customizing these properties to meet tissue-specific requirements is essential for successful organoid culture. The strength of ECM hydrogels can be enhanced by incorporating substances such as PEGDA, which interacts with ECM components to produce smaller, more uniform pores. Alginate, a biocompatible polysaccharide, improves mechanical properties and facilitates hydrogel formation through ionic cross-linking. Adding ECM components such as collagen and fibronectin can further refine the hydrogel’s topological and mechanical characteristics (Table 3).

**Table 3:** Modified hydrogel was used as culture substrates

Source	Addition	Cultivate	Extracellular matrix component source	Enhancement aspect	References
Plant	Alginate	Mouse preovarian follicle	Human and bovine ovarian	Oocyte maturation rate was significantly improved	[117]
	Agarose	Patient-derived bone MSCs and CDCs	Patient’s cartilage	The proliferation rate of CDC and MSC was increased	[118]
Animal	Hyaluronic acid and collagen	Patient-derived sarcoma organoids	Patient’s sarcoma	Providing a more tumor-like environment in vivo facilitates drug screening	[119]
	Fibrinogen	Rat neonatal cardiomyocytes	Pig ventricular	Better spontaneous heartbeat recovery, frequency, synchronization and maintenance	[113]
Synthesis	Epoxyeicosatrienoic acid (EET)	Rabbit esophageal epithelial cells	Pig esophagus	The metabolic activity of rabbit epithelial cells was improved	[120]
	Hyaluronic acid methacrylate (HAMA)	Mouse pancreatic cells	Mouse pancreatic	Maintain the adhesion and morphology of islet cells and improve islet function and activity	[121]

Modifying hydrogel mechanics is also vital in replicating the morphology of tissues and organs under pathological conditions. For example, the stiffening of liver and lung tissues following lesions can be simulated by adjusting hydrogel properties, thus enabling the development of realistic organoid models for studying differentiation, culture dynamics, and drug responses [77, 116]. The mechanical environment, as demonstrated by the optimal growth of intestinal organoids on hydrogels with specific elastic moduli, plays a significant role in organoid development [112].

### Bioactive substances in hydrogels

The previously outlined attributes of ECM hydrogels primarily emphasize their physical and chemical properties, highlighting their efficacy as scaffolding materials. Nonetheless, it is crucial to recognize that glycosaminoglycans, proteoglycans, and cytokines embedded within the extracellular matrix are also critical to the functionality of ECM hydrogels. Various glycoproteins have been shown to play significant roles in both normal tissue physiology and the maintenance of tissue homeostasis, as well as in adaptive responses to perturbations such as mechanical loading/unloading or tissue injury followed by regeneration [122–124]. Furthermore, alterations in glycoprotein expression are commonly observed in pathological conditions such as cancer, fibrosis, or connective tissue disorders.

As integral components of the ECM, GAGs and aminoglycans have the capacity to bind, anchor, and release cytokines in response to specific stimuli. Consequently, ECM hydrogels can provide organoids with an environment that effectively mimics the in vivo microenvironment. Moreover, it is possible to customize hydrogels by modulating the concentration and characteristics of bioactive molecules to meet specific requirements [125]. The types and concentrations of cytokines are closely linked to the destiny of organoids; hence, incorporating diverse types and concentrations of cytokines into hydrogels can significantly influence cellular outcomes. Variations in bioactive molecules within the ECM of different tissues are responsible for the distinct effects exhibited by prepared hydrogels on the same type of organ [126].

## ECM hydrogels with organoid culture

### Isolation of organoids and their routine culture

Organoids are functional in vitro cell culture models designed to replicate the in vivo environment. Simultaneously, organoids facilitate the in vitro growth of cells within a 3D environment, leading to the formation of small cell clusters that undergo self-organization and differentiation into functional cell types, thus recapitulating both the structure and function of bodily organs. The development of functional organoids depends not only on the intrinsic ability of cells to self-assemble into a 3D architecture resembling native organs but also on exogenous components such as differentiation factors and the ECM [127].

In 1907, Henry Van Peters Wilson demonstrated the capacity of dissociated sponge cells to undergo self-organization and regenerate complete organisms. Since 1981, stem cell research has witnessed a remarkable surge following the successful isolation and generation of pluripotent stem cells (PSC) from mouse embryos [128]. The recent advancement in the field of induced pluripotent stem cells (iPSCs), which can be generated through reprogramming of mouse and human fibroblasts, has significantly revolutionized research on stem cells and organoids [129]. Nowadays, organoids can be derived from pluripotent stem cells (PSCs), including embryonic stem cells (ESCs) and induced pluripotent stem cells (iPSCs), as well as organ-specific adult stem cells (ASCs).

The establishment of a 3D culture system for organoids represents a crucial milestone, which can be achieved through either scaffold or scaffold-free techniques. Scaffolds primarily provide structural support and ECM signals, with Matrigel being the most commonly used option due to its heterogeneous gelatinous protein mixture secreted by Engelbreth–Holm–Swarm (EHS) mouse sarcoma cells, consisting mainly of adhesion proteins such as collagen, internal actin, laminin and heparin sulfate proteoglycan [130]. Scaffold-free techniques can be established using the “gas–liquid interface,” where cells are cultured on a basal layer of fibroblasts or matrix glue initially immersed in a medium that gradually evaporates, thereby exposing the upper layer of cells to air [131]. Additionally, inverted culture methods can also be employed.

The integration of organoids within the ECM system is a multifaceted process that begins with the self-assembly of cells into a three-dimensional structure. This process is facilitated by the ECM’s ability to provide both structural cues and bioactive signaling molecules that guide organoid morphogenesis and function. For instance, the stiffness of the ECM can influence the differentiation of stem cells into organ-specific cell types, while the presence of growth factors can promote cell proliferation and survival. Additionally, the ECM’s porous structure allows for the diffusion of nutrients and waste products, which is essential for the maintenance of organoid viability. As research progresses, advanced techniques such as 3D bioprinting and microfluidics are being employed to create more complex and functional organoid-ECM systems, offering promising avenues for modeling diseases and testing therapeutics.

Currently, organoids are widely used in development and disease modeling, precision medicine, toxicology research, and regenerative medicine. Organoids can be combined with new technologies to highlight their unique advantages, using gene editing tools (such as gene transfer, CRISPR–Cas9, or RNA interference methods) to introduce pathological mutations into wild-type organoids to design human cancers [132]; large-scale collection of organoids from patients is helpful for organoid database construction and drug toxicity evaluation. In the future, it also has great potential as a tool to save patients with organ failure. Existing studies have shown that mouse colonic organoids can indeed be expanded and transplanted into damaged mouse colons to form functional crypt units, and PSC-derived liver organoids can also save liver failure in mouse liver injury repair [133].

To summarize, organoids represent a promising model; however, several limitations and challenges persist. Many organoids are cultivated in Matrigel, which contains tumor components, while general organoids are grown in medium saturated with growth factors. This excessive presence of growth factors surrounding the organoids may compromise the natural morphogen gradients of the tissues. Simultaneously, cost considerations must also be taken into account. Therefore, it is imperative to urgently identify a more stable and cost-effective culture matrix for promoting widespread application of organoids.

The methodology for isolating and culturing organoids from diverse tissues is similar, and the cells can be prompted to form organoids by cultivating them in Matrigel enriched with suitable exogenous factors, such as chemical small molecule inhibitors/activators, cytokines, and media supplements. Although the process of organoid isolation and procurement is similar, the culture phase varies significantly, and even for tissues with structurally similar features, such as the small intestine and colon, the requisite reagent cocktail for organoid preparation varies. Therefore, when formulating hydrogels for organoids, it is imperative to consider the cells’ native living environment and the variety and quantities of various factors that must be incorporated during their sustained culture as organoids.

However, with the ongoing advancements in decellularization technology, the capability to procure the in situ extracellular matrix of target organoids has become attainable. To a certain extent, the issues associated with xenogeneic or heterologous cytokines influencing organoids cultured with commercial gelatin have been addressed [134]. Concurrently, the decellularization technique maintains the compositional disparities observed between basal and apical regions, thereby underscoring the advantage of culturing corresponding organoids with hydrogels derived from the natural biomaterials’ extracellular matrix [135]. Furthermore, the extracellular matrix can effectively retain an array of bioactive substances, including cytokines and glycosaminoglycans, which also furnishes a viable experimental concept and foundation for the in vitro emulation of organ or cell interactions from disparate sources [136].

### Organoid culture on non-commercial hydrogels

The commercial hydrogel Matrigel, widely utilized for organoid culture, is derived from components sourced from murine tumors. However, the microenvironment it provides for organoids in vitro significantly deviates from the in vivo setting due to the complex nature of the extracellular matrix. Variations in tissue properties, such as elasticity and toughness, are significant indicators of mechanical support disparities. Additionally, each tissue performs distinct functions, further accentuating variations in bioactive substances. Cultivating organoids on hydrogels prepared with tissue-specific matrices, rather than commercial matrix adhesives, often yields superior outcomes such as enhanced preservation of differentiation potential, accelerated proliferation rates, improved organ-like functionality, and earlier expression of maturity.

**Fig. 4:**
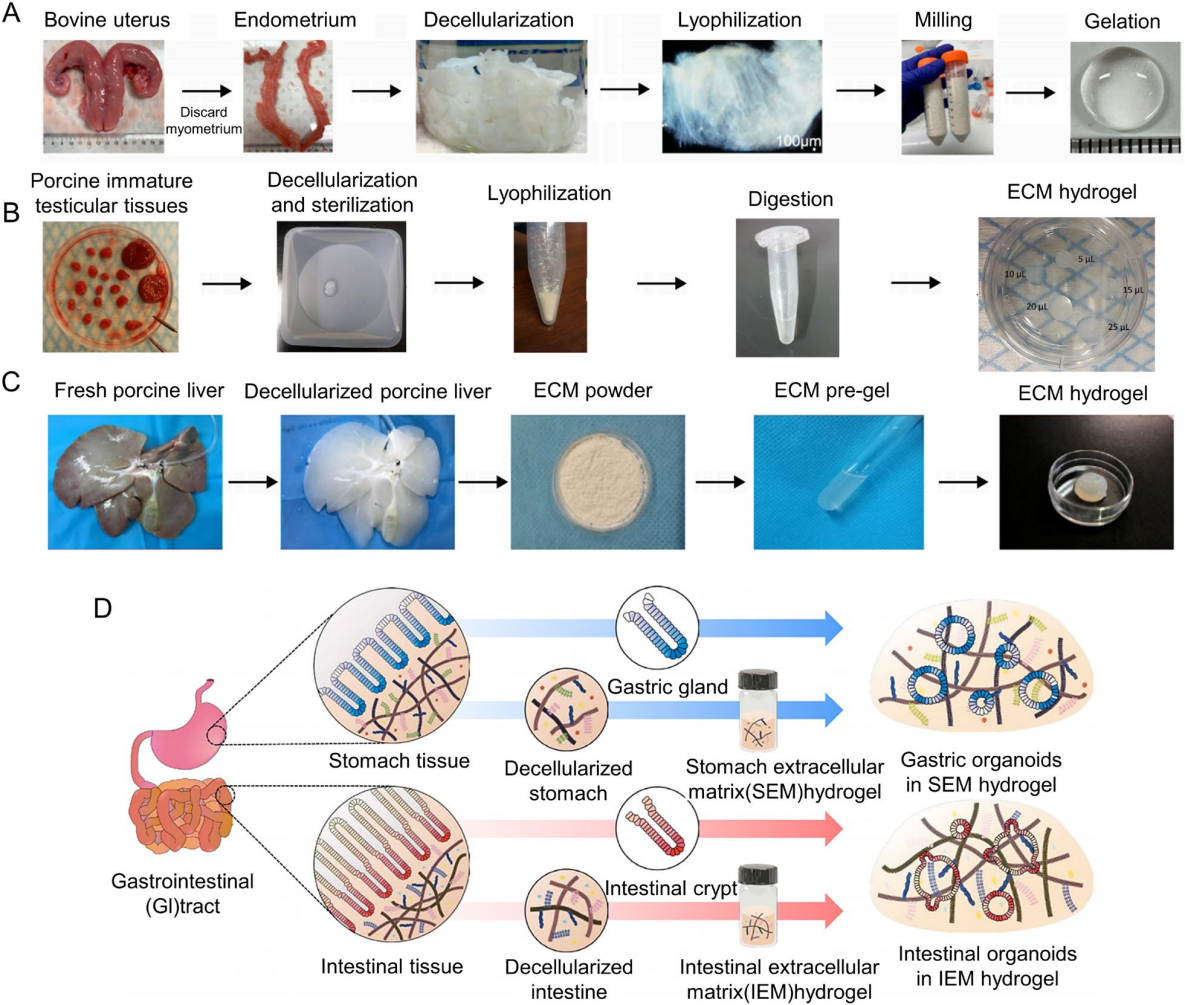
**A** Overview of the key steps involved in endometrial hydrogel preparation from the bovine endometrium [137]. (Copyright © 2022 the Author(s). Published by PNAS). **B** Porcine immature testicular tissues (ITTs) were dissected in small fragments and decellularized before being lyophilized and digested in a solution of HCl/pepsin (n = 20). Drops of 5, 10, 15, 20 and 25 µL were incubated for 1 h at 34 °C to evaluate manipulability after gelation [138]. (Copyright © 2019 by the authors). **C** Procedure for the formation of porcine liver extracellular matrix (PLECM) gels [139]. (Copyright ©The Author(s) 2020. Published by Baishideng Publishing Group Inc. All rights reserved). **D** Schematic illustration of the generation of gastrointestinal (GI) organoids using ECM hydrogels SEM, IEM. [Decellularized stomach derived ECM (SEM) decellularized intestine derived ECM (IEM)] [140]. (Copyright © The Author(s) 2022)

Figure 4 shows several examples of the process from organizing to obtaining the corresponding hydrogel. Jamaluddin et al. ‘s hydrogel preparation protocol is divided into five key steps: (1) surgical separation of the endometrium, (2) removal of cellular and nuclear material (decellulated), (3) lyophilization or freeze–drying, (4) low-temperature grinding to powder, and (5) digestion, pH neutralization, and gelation (Fig. 4A) [137]. In their experimental steps, special emphasis was placed on the importance of low temperature grinding, which is the key to efficient enzymatic hydrolysis. Vermeule et al. cut pig immature testicular tissue (ITTs) into small pieces for decellularization, which increased the efficiency of decellularization to a certain extent (Fig. 4B) [138]. He et al. used SDS and Triton X-100 to inject the whole liver from the portal vein to obtain extracellular matrix because the liver is rich in blood vessels, which is conducive to the action of acellular reagents (Fig. 4C) [139]. In order to explore a better decellulatory process, Kim et al. evaluated two decellulatory methods and found that for gastrointestinal tissue, the use of ionic detergents (e.g., sodium deoxycholate; although the decellularization regimen of SDC effectively removed the cell components, the retention of extracellular matrix was reduced, and the GAG content in ECM was significantly reduced. An optimized decellulatory regimen, based on a non-ionic detergent (Triton X-100), can completely remove the cellular component from stomach and intestinal tissues, while the major ECM components (e.g., glycoaminoglycans; GAG) is retained. Figure 4D shows the flow of gastrointestinal tissue from decellularization to hydrogel production [140].

Organoid culture after the successful preparation of ECM hydrogels is crucial. Compared with the basal membrane matrix, many ECM-derived hydrogels exhibit the same level of organoid culture as the basal membrane matrix, and may have more outstanding performance in some aspects. Choi et al. cultured patient-derived lung cancer organoids (LCOs) in parallel with dECM hydrogels from lung tissue and Matrigel and found that LCOs in dECM hydrogels were more prone to long-term proliferation (over 7 days). Specifically, the 14-day proliferation rate of LCOs in dECM hydrogel was significantly different from that of LCOs cultured in Matrigel, and the LCOs in dECM retained the genetic changes of the original cancer tissue [141]. Although the hydrogel prepared by Simsa et al. from ECM of adult pig brain cells lacked certain brain-specific proteins, its efficacy in promoting human brain organoid culture was comparable to that of Matrigel [142]. These findings highlight the potential for using normal pig or human tissue as a commercial mouse tumor hydrogel replacement, thereby mitigating potential adverse effects.

The morphology of organoids in hydrogels is also an important aspect to evaluate organoids. The hydrogel extracted from dECM of bovine lung tissue by Kuzieoglu et al. successfully retained the morphology of patient-derived lung organoids, providing a reproducible analog for human lung tissue suitable for disease modeling [143]. Willemse et al. successfully propagated intrahepatic bile duct cell organoids (ICOs) in ECM hydrogels of pig and human livers, showing stable proliferation and generating functional bile duct shaped organoids [144].

**Fig. 5:**
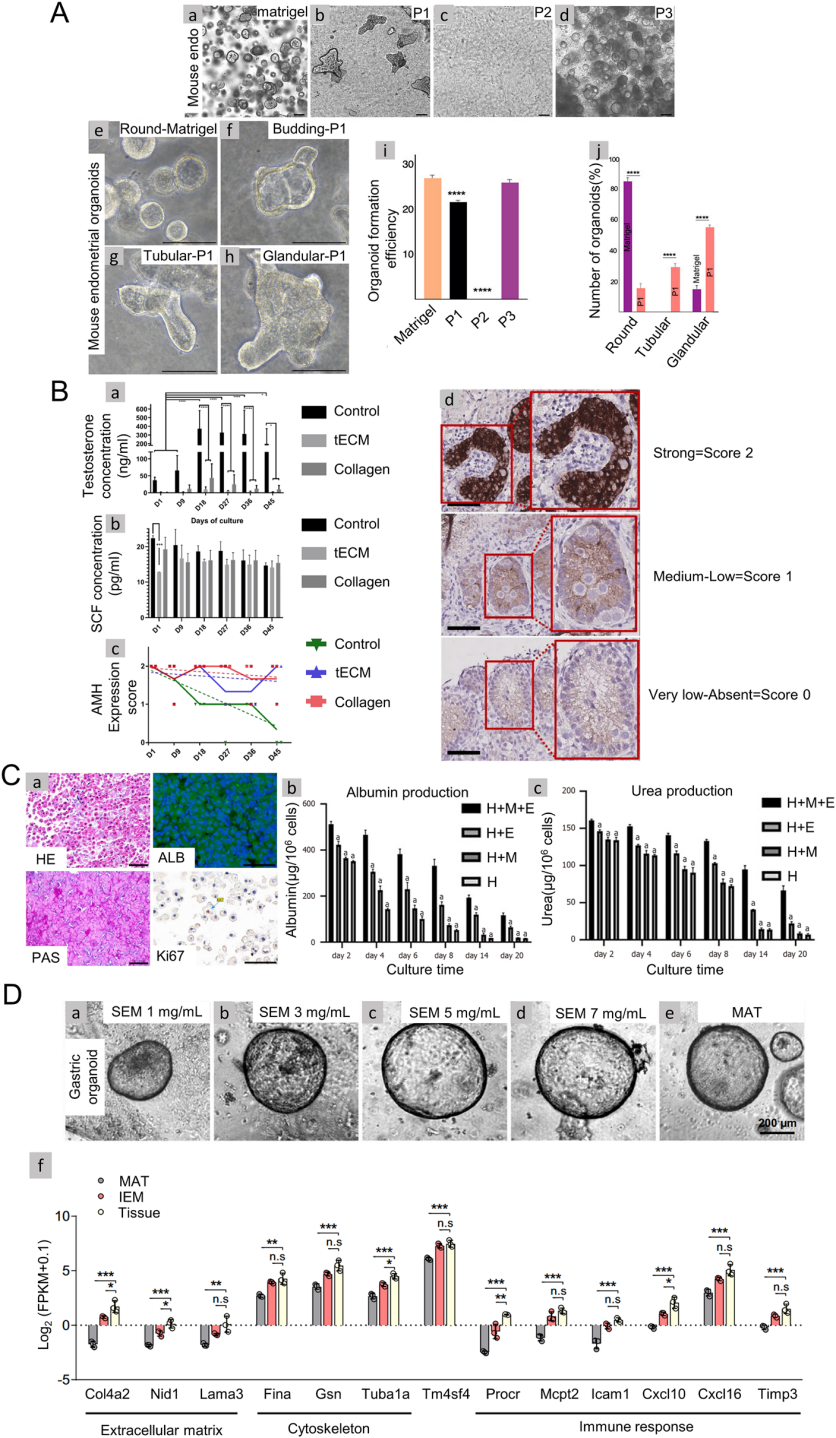
**A**, **a**–**d** Bright-field images of mouse endometrial organoids in Matrigel and endometrial hydrogels. The organoids were cultured with hydrogels produced by tissues treated with three different detergents: 4% SDS (hereinafter referred to as P1), 1% SDS (P2) and 4% SDC (P3). **A**, **e** Shows the round-shaped mouse endometrial organoids, which was cultured in Matrigel (**A**, **e**). In comparison to circular organoids embedded in Matrigel, the organoids cultured in P1 hydrogel exhibited morphological characteristics resembling budding (**A**, **f**), tubular (**A**, **g**), and glandular structures (**A**, **h**) similar to those observed in mouse endometrial tissue. **A**, **i** The organoid forming efficiency of mouse endometrial organoids in Matrigel and P1–P3 hydrogel. **A**, **j** Percentage of formation of round, tubular and glandular mouse endometrial organoids in Matrigel and P1 hydrogel [137]. (Copyright © 2022 the Author(s). Published by PNAS). **B** Evaluation of Leydig cells (LC) and Sertoli cells (SC) functionality and maturation in control tissue and TOs. The authors set up three groups of experiments, with normal tissue slices and supernatant collected in vitro culture as the control group (Control). Testicular ECM hydrogels (tECM) prepared using acellular porcine immature testicular tissue (ITT) scaffolds were used as a group. There was also the Collagen hydrogel group (Collagen), which consists primarily of type I collagen but also contains small amounts of other types of collagens (II, III, V, and VI). **B**, **a**, **b** Testosterone and stem cell factor (SCF) was quantified in culture supernatants. **B**, **c** Maturation of SCs was monitored in control tissue and TOs by immunohistochemistry (IHC) for anti-Mullerian hormone (AMH). Maturation of SCs evaluated using a score based on AMH immunostaining demonstrated a significant decrease over time in control but not in tECM and collagen groups. **B**, **d** Representation of the scores used to determine AMH intensity staining [138]. (Copyright © 2019 by the authors). **C**, **a** Hematoxylin and eosin (HE) staining, periodic acid-Schiff (PAS) staining, immunofluorescence staining for albumin (ALB; green), and immunohistochemical staining for Ki67. The nuclei were counterstained with DAPI (blue). **C**, **b** Secretion of albumin was measured by ELISA on days 2, 4, 6, 8, 14, and 20; **C**, **c** Urea synthesis was measured on days 2, 4, 6, 8, 14, and 20. H + M + E group: hepatocytes and MSCs seeded on ECM-gel pre-coated plate; H + E: hepatocytes seeded on ECM-gel pre-coated plate; H + M: hepatocytes and MSCs seeded on ECM-gel free plate; H group: hepatocytes seeded on ECM-gel free plate; ECM: extracellular matrix [139]. (Copyright ©The Author(s) 2020. Published by Baishideng Publishing Group Inc. All rights reserved). **D**, **a**–**e** Brightfield images of gastric organoids grown in decellularized stomach derived ECM (SEM) hydrogels and Matrigel (MAT) at day 5. **D**, **f** Comparison of expression values (log_2_ [FPKM + 0.1]; FPKM, fragments per kilobase of transcript per million mapped reads) of selected genes involved in **f** gastric or **g** intestinal development and homeostasis in native gastrointestinal (GI) tissues and in GI organoids cultured in GI tissue-derived ECM hydrogels or Matrigel [140]. Core matrisome protein-encoding genes (Col4a2, Nid1, and Lama3), cytoskeleton-related genes (Flna, Gsn, and Tuba1a), intestinal epithelial gene (Tm4sf4), immune response (Procr, Mcpt2, Icam1, Cxcl10, Cxcl16, and Timp3). (Copyright © The Author(s) 2022)

In addition, it is important to evaluate the suitability of hydrogels for organoid culture. Jamaluddin et al. prepared hydrogels from endometrial ECM and assessed their quality based on organoid formation efficiency, morphology, and size (Fig. 5A). After P1 hydrogel was selected, the endometrial gland marker Foxa2 and proliferation marker Ki67 in the organoids were immunostained and quantified, and it was confirmed that P1 hydrogel could effectively replace matrix gel culture of endometrial organoids [137]. Vermeule et al. found that testicular cells isolated from pig immature testicular tissue (ITT), when cultured in vitro in a hydrogel, can form testicular organoids (TOs) with tubular structures similar to those of natural organs. Three conditions were examined in that study: testosterone and stem cell factor (SCF) in supernants during culture of acellular ITT, testis ECM hydrogel (tECM) group, and collagen hydrogel group (collagen) group, while anti-Mullerian hormone (AMH) in sections was detected using immunohistochemical techniques to assess the maturity of Sertoli cells (SC) in testicular tissue and TOs. Immunohistochemical scores based on AMH showed a significant reduction in SC maturity over time in the control group, but not in the tECM and collagen groups, suggesting that TOs mimics some physiological aspects of testicular function, but with significant differences in hormone production and cell maturity (Fig. 5B). It is suggested that the composition of culture environment can be further explored and its mechanical properties can be changed to provide a more suitable three-dimensional environment for culture [138]. Hepatocyte organoids developed by He et al. through co-culture with MSCs on PLECM gel have been shown to maintain hepatocyte function and prolong viability for at least 20 days (Fig. 5C), as demonstrated by the detection of liver-specific markers ALB and Urea [139]. Okada et al. successfully promoted the differentiation of neonatal mouse testicular cells into testicular organoids (TOs) on the ECM hydrogel of ram testis, confirming the progression of spermatogonium into the post-meiosis stage [145]. Kim et al. day 5 organoids grown in acellular gastro-derived ECM (SEM) hydrogels and Matrigel (MAT) (Fig. 5D, a–e). In addition, SEM and IEM hydrogels promoted the colonization of transplanted organoids in damaged gastrointestinal (GI) tissues. The expression of some genes involved in wound healing was upregulated in GI ECM organoids compared to basal membrane matrix organoids (Fig. 5D, f), suggesting that organoid transplantation using GI tissue-derived ECM hydrogels has the potential to provide a highly effective therapeutic approach [140].

These advancements demonstrate the multiple functional applications of organoids and contribute to the progress of tissue engineering technology. The application potential of extracellular matrix hydrogels is highlighted by summarizing several articles on organoids cultured on hydrogels derived from the extracellular matrix in Table 4. For patient-derived organoids, utilizing a hydrogel prepared from the extracellular matrix of the same type of tissue for culture can yield significant proliferation results. The drug sensitivity and growth rate of patient-derived organoids cultured with the same type of tissue hydrogels surpass those cultured with commercial hydrogels, thereby illustrating that the composition of the extracellular matrix is closely related to the fate of organoids.

**Table 4:** Extracellular matrix hydrogel culture organoids

Organoid type	Source of extracellular matrix hydrogel	Enhancement aspect	References
Patient-derived vascularized LCOs	Porcine lung	The proliferation of fibrotic LCOs cells and the expression of drug-resistance related genes were increased	[141]
Patient-derived lung organoids	Bovine lung	Patient-derived lung organoid morphology was preserved in ECM hydrogels	[143]
ICO	Human or porcine livers	The proliferation of choanocytes organoids and the choanocytes-like phenotype can be maintained	[144]
Human brain organoids derived from hESC	Porcine brain	Human brain organoids can grow normally, and it is speculated that some proteins can be added to the hydrogel to promote its proliferation	[142]
Mouse and human endometrial organoids	Human and bovine endometrium	Endometrial organoids are proteomically closer to natural tissues	[137]
Porcine TOs	Porcine ITT	By examining stem cell factor and testosterone, it was found that SCs had enhanced function and better potential to restore the reproductive capacity	[138]
Mouse liver organoids constructed from human MSCs and mouse primary hepatocytes	Porcine liver	Generated primary hepatocyte organoids fast; primary hepatocellular organoids can survive for a long time and maintain stable function	[139]
Mouse TOs	Ram testis	Multicellular TOs can be produced, confirming the differentiation of spermatogonial cells into postmeiotic cells	[145]
Mouse GI organoids	Porcine stomach and intestine	Several genes involved in wound healing are upregulated in gastrointestinal organoids, which enable better cell component biogenesis and structural development	[140]
hiPSC-EB layered retinal organoids	Porcine sclera and uvea	Promotes hiPSC-EBs morphogenesis and neural/retinal differentiation, cambium formation of layered retinal organoids	[146]
Human endometrial organoids	Porcine endometrium	Organoids not only grew and recapitulated the molecular and functional characteristics of the tissue of origin, but also showed a higher proliferation capacity than those cultured by previously described techniques	[111]
Kidney organoids derived from hPSCs	Porcine kidney	The kidney organoids have more mature patterns of glomerular development and higher similarity to human kidney	[73]
Liver organoids generated by self-organization of human hepatocarcinoma cells together with human mesenchymal and endothelial cells	Sheep liver	Enhanced the functional activity of self-organized liver organoids	[147]

## Conclusion and perspective

Organoid culture is deeply influenced by its microenvironment, and the transition from 2 to 3D culture was designed to enhance the fidelity of organoid models to in vivo conditions. Initially, products like Matrigel and BME adhesive were crucial in the nascent stages of organoid culture. However, as our understanding and applications of organoids have evolved, the origin and composition of these adhesives have increasingly impacted experimental outcomes. With advancements in cellular technology, the unique value of hydrogels derived from cellular scaffolds in organoid culture has become widely recognized. Consequently, the development and application of hydrogels prepared from acellular scaffolds have emerged as a current focal point in this field.

Nevertheless, to accommodate diverse organoid types and research objectives, modifications to existing foundational hydrogels are necessary. This review explores the current methodologies for ECM hydrogel preparation, aiming to broaden the research and application scope of organoids across various models and research domains. Currently, the application of acellular scaffold hydrogels in organoid culture is somewhat limited due to the absence of standardized criteria for evaluating these scaffolds and hydrogels, and the lack of uniform protocols for their preparation. These constraints impede further research and applications of cellular scaffold hydrogels in organoid culture.

The establishment of formal criteria for assessing the purification level during scaffold fabrication and evaluating the physical and chemical properties of foundational hydrogels—including turbidity, rheological properties, morphological analysis, swelling degradation profiles, and chemical composition analysis—is imperative. Enhancing these standards will facilitate the expansion of clinical implementation of the organoid model. Moreover, investigating the stability of hydrogels after the introduction of exogenous ingredients is crucial. Determining whether the incorporation of ingredients from various sources yields distinct impacts on organoid culture is a worthwhile endeavor.

In summary, hydrogels derived from cellular scaffolds have demonstrated unique advantages in organoid cultivation. The ongoing development of composite hydrogels holds promise for establishing a more physiologically relevant environment for organoids, beneficial for disease modeling, tissue regeneration, and clinical research. While technical challenges remain in achieving tailored production of hydrogels through targeted modifications of cellular scaffolds, the progress in this field is compelling. This model not only offers a more authentic representation of organoid-like cells but also contributes to the advancement of acellular scaffolds—a natural biomaterial—in technologies such as 3D printing and microfluidics cell culture.

## Data Availability

Not applicable.

## Abbreviations

ECM: Extracellular matrix
3D: Three-dimensional
2D: Two-dimensional
BME: Basement membrane extract
dECM: Decellularized ECM scaffolds
IVD: Intervertebral disc
GAGs: Glycosaminoglycans
scCO2: Supercritical carbon dioxide
GI: Gamma irradiation
EI: Electron irradiation
EtO: Ethylene oxide
DMSO: Dimethyl sulfoxide
MSCs: Marrow-derived mesenchymal stem cells
CDCs: Cartilage derived cells
EET: Epoxyeicosatrienoic acid
HAMA: Hyaluronic acid methacrylate
LCOs: Lung cancer organoids
ICO: Intrahepatic cholangiocyte organoids
iPTOs: Immune-enhanced patient tumor organoids
hiPSCs: Human induced pluripotent stem cells
hiPSC-EB: Embryoid derived from human induced pluripotent stem cells
hESC: Human embryonic stem cells
MSCs: Mesenchymal stem cells
TOs: Testicular organoids
ITT: Immature testicular tissue
SCs: Sertoli cells
hPSCs: Human pluripotent stem cells
GI: Gastro-intestinal
